# Small area variations in four measures of poverty among Indian households: Econometric analysis of National Family Health Survey 2019–2021

**DOI:** 10.1057/s41599-023-01509-0

**Published:** 2023-01-17

**Authors:** Anoop Jain, Sunil Rajpal, Md Juel Rana, Rockli Kim, S. V. Subramanian

**Affiliations:** 1 Global Health & Social Medicine, Harvard Medical School, Boston, MA 02115 USA; 2 Interdisciplinary Program in Precision Public Health, Department of Public Health Sciences, Graduate School of Korea University, Seoul, South Korea; 3 Department of Economics, FLAME University, Pune, India; 4 G B Pant Social Science Institute, Prayagraj, India; 5 Division of Health Policy & Management, College of Health Science, Korea University, 145 Anam-ro, Seongbuk-gu, Seoul, 02841 South Korea; 6 Harvard Center for Population and Development Studies, Cambridge, MA 02138 USA; 7 Department of Social and Behavioral Sciences, Harvard T.H. Chan School of Public Health, Boston, MA 02115 USA

**Keywords:** Development studies, Health humanities

## Abstract

India has seen enormous reductions in poverty in the past few decades. However, much of this progress has been unequal throughout the country. This paper examined the 2019–2021 National Family Health Survey to examine small area variations in four measures of household poverty. Overall, the results show that clusters and states were the largest sources of variation for the four measures of poverty. These findings also show persistent within-district inequality when examining the bottom 10th wealth percentile, bottom 20th wealth percentile, and multidimensional poverty. Thus, these findings pinpoint the precise districts where between-cluster inequality in poverty is most prevalent. This can help guide policy makers in terms of targeting policies aimed at reducing poverty.

## Introduction

Income and wealth are measures of socioeconomic position (SEP) that have long been connected to health outcomes through myriad pathways and mechanisms (Adler et al., 1994; Braveman and Gottlieb, 2014; Galobardes et al., 2007; Oakes and Kaufman, 2006; S. V. Subramanian et al., 2002). Impoverished parents are often unable to provide children with adequate nutrition, safe drinking water, or improved sanitation (Karlsson et al., 2020; Victora et al., 2003). Poor households are also more likely to be in areas that lack access to healthcare, food security, and centralized waste management (Mosley and Chen, 1984; Victora et al., 2003), and are more vulnerable to the effects of climate change (Hallegatte and Rozenberg, 2017), which further exacerbates deleterious health outcomes (McMichael et al., 2007; Romanello et al., 2021). Poverty is also associated with adverse mental health outcomes (Lund et al., 2010; Patel and Kleinman, 2003).

In India, the Global Multidimensional Poverty Index found that 271 million Indians were lifted out of poverty between 2006 and 2016 (Initiative et al., 2019). However, much of this progress has been geographically varied throughout the country. For example, while national data show a falling poverty headcount ratio between 1983 and 1994, states such as Assam, Haryana, and Himachal Pradesh experienced increases (Himanshu, 2007). Additionally, while Andhra Pradesh experienced the greatest decline in multidimensional poverty between 1999 and 2006, Bihar’s reduction was the slowest during the same period (Alkire and Seth, 2015). Other studies have examined India’s 88 regions defined by the National Sample Survey Organization according to climate, language, and culture (Chauhan et al., 2016). While some of these regions, such as Tamil Nadu and Karnataka, experienced significant declines in poverty between 1993 and 2012, other regions in southern Odisha and Chhattisgarh continue lagging behind (Chauhan et al., 2016). Districts have also been targeted with poverty eradication policies, such as investing in industrial and agricultural growth, given significant inter-district disparities within states (Chandra, 2021; Chaudhuri and Gupta, 2009).

However, single-level analyses assume a certain degree of homogeneity within a given geography despite evidence pointing to significant intra-unit inequalities (Kapur Mehta and Shah, 2003; Singh et al., n.d.). Varying agricultural and ecological conditions, for example, are associated with disparate agricultural yields and thus poverty rates within states (Palmer-Jones and Sen, 2003, 2006). These within-region variations can be seen when looking at certain health outcomes, such as child malnutrition, which is one indicator in the Multidimensional Poverty Index (MPI) (Initiative et al., 2019). A recent study showed that 93% of the variation in child stunting (height-for-age *Z* score), an anthropometric indicator of malnutrition, is attributable to between-individual variations (Mejía-Guevara et al., 2015). Similarly, 80–85% of the variation in child undernutrition was attributable to within-population differences in India (Mejía-Guevara et al., 2015; Rodgers et al., 2019). Such evidence points towards the importance of considering variation within geographical units, such as districts, while designing targeted strategies under maternal and child nutrition programs.

Similar types of within-population analyses of poverty throughout India have not been done. For example, the Indian government launched the Aspirational Districts Program (ADP), an initiative targeting the 112 least developed districts (Porter and Stern, n.d.). While this program targets poverty eradication programs at the district level, it does not take into account the variations in poverty that might exist within districts and between communities. Understanding these small area variations in poverty is important given that previous research has shown how child malnutrition and dietary diversity, indicators of poverty, also vary significantly within districts and between clusters (Jain et al., 2022; Rajpal et al., 2021).

Given this background, the purpose of this paper was to better understand within-district and between-community variations in poverty in order to inform the effective targeting of poverty-eradication policies throughout India. Doing so is important considering that as per the most recent census data from 2011, almost 22% of people in India lived on less than USD 1.90 per day (GoI 2011 Census of India, 2011). Therefore, we examined these variations using four different measures of household poverty. These were (a) bottom 10th wealth percentile; (b) bottom 20th wealth percentile; (c) below the poverty line; and (d) the multidimensional poverty index. We used data from the fifth round of the National Family Health Survey (NFHS) from 2019 to 2021.

## Methods

### Data source and sample

**Fig. 1:**
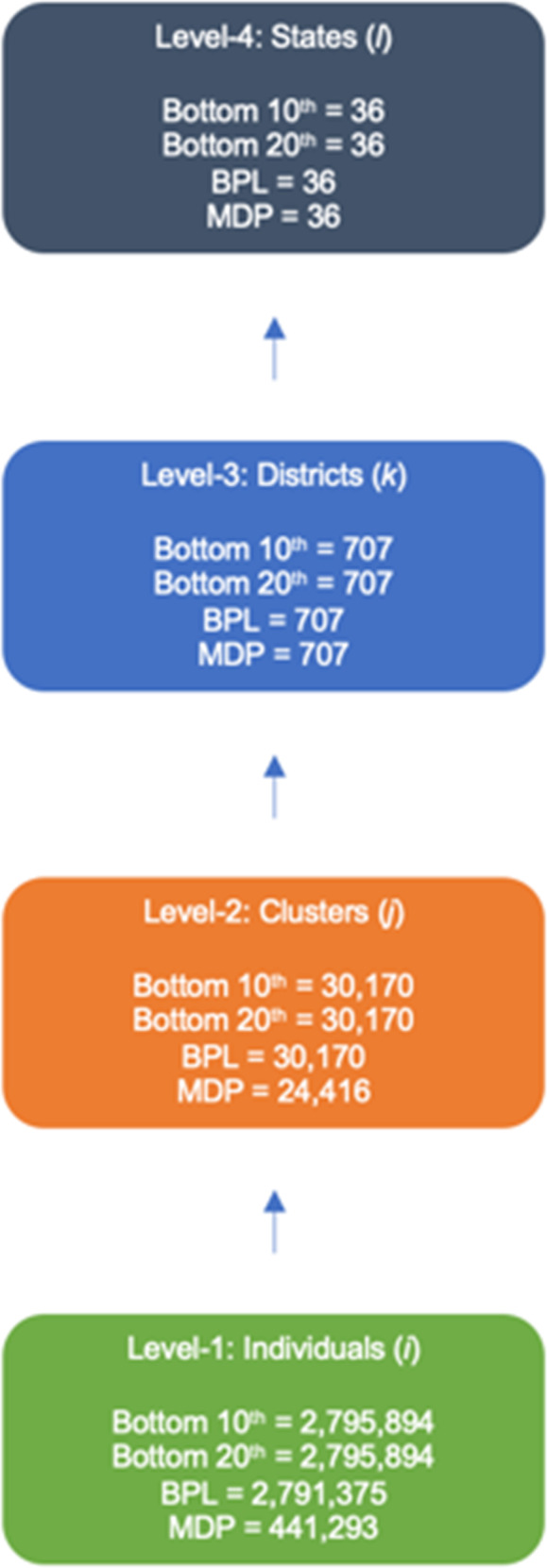
Multilevel structure for four measures of poverty across states and Union Territories in India. Green describes individuals, orange describes clusters, blue describes districts, and gray describes states.

This analysis was conducted using data from the fifth round of the National Family Health Survey (NFHS). These data were collected between 2019 and 2021. A two-stage cluster sampling strategy was employed for household selection. The primary sampling units (PSUs) were clusters, defined as groups of adjacent households. The first stage of sampling involved selecting rural and urban clusters. Clusters containing more than 300 households were divided into smaller groups from which households were selected in the second stage of sampling. No more than 22 households were selected from any given PSU. The NFHS includes data from 2,795,894 de jure household members, nested in 30,170 rural and urban clusters, in all 707 districts, and in all 36 states/union territories. The multilevel structure for the four measures of poverty is presented in Fig. 1.

### Primary outcomes

We analyzed the small area variation of the percent prevalence of individuals in the bottom wealth decile and bottom wealth quintile. These ranges are constructed by assigning each member of the household the wealth index score. Individuals are then ranked against the entire population based on their scores. This distribution is then divided into equal bins. Both of these outcomes were dichotomized such that individuals were either in the bottom 10th or 20th percentiles, or above.

We also analyzed the small area variation of individuals that have a below-poverty line (BPL) card. These cards are distributed to poor households by state governments, entitling households to 25–35 kg of subsidized grain per month as per state government guidelines. Individuals in BPL households were considered BPL for this study.

Finally, we analyzed the small area variation of multidimensional poverty (MDP). The MDP captures deprivations across three dimensions, health, education, and standard of living. Under health, the MDP includes indicators of nutrition, mortality, and antenatal care. Under education, the MDP includes indicators of years of schooling and current school attendance. Under the standard of living, the MDP includes the type of cooking fuel, sanitation, drinking water, electricity, housing quality, assets, and bank account. We used these indicators to construct a deprivation score following the weighting process outlined in the NITI Aayog MDP baseline report (India National Multidimensional Poverty Index, 2021). Households with a score >0.33 are considered multidimensionally poor. Individuals in MDP households were considered MDP for this study.

### Statistical analysis

The NFHS data are structured such that individuals at level one were nested in clusters at level two, districts at level three, and states at level four. Each of the outcomes included in our analysis was binary. Therefore, we estimated four four-level variance component models to decompose the proportion of geographic variation attributable to clusters, districts and states for individual *i* in cluster *j*, district *k*, and state *l* using Eq. (1)

1 $${\rm {logit}}( {\pi _{ijkl}} ) = \beta _0 + ( {u_{0jkl} + v_{0kl} + f_{0l}} )$$

In this model, *π*_*ijkl*_ is the log odds of the outcome for individual *i*. The random effects are the residual differentials for clusters (*u*_0*jkl*_), districts (*v*_0*kl*_), and states (*f*_0*l*_). Each of the residual differentials is assumed to be normally distributed with a mean of zero and a variance of *u*_0*jkl*_ ~ N(0, $\sigma _{u0}^2$), *v*_0*kl*_ ~ N(0, $\sigma _{v0}^2$), and ***f***_0*l*_ ~ N(0, $\sigma _{f0}^2$) where the variances quantify the between-cluster, between-district, and between-state variation, respectively. The variance at level one (households) cannot be computed in models with binary outcomes (Kim et al., 2016).

The proportion of variation attributable to each geographic level—clusters, districts, and states—was calculated by dividing the variance of a given level by the total geographic variation (i.e., for the cluster level, $\sigma _{u0}^2$/($\sigma _{u0}^2$ + $\sigma _{v0}^2$ + $\sigma _{f0}^2$) × 100). We conducted this analysis in MLwiN 3.05 using the Monte Carlo Markov Chains method with a burn-in of 500 cycles and monitoring of 5000 iterations of chains, the same procedure used in previous studies (Jain et al., 2022; Rajpal et al., 2021).

Next, we generated precision-weighted estimates specific to each cluster for each outcome. This was done using Eq. (2)

2 $${\mathrm {exp}} \lbrack\beta _{0} + ( {u_{0jkl} + v_{0kl} + f_{0l}})\rbrack/ 1+{\mathrm {exp}} \lbrack\beta _{0} + ( {u_{0jkl} + v_{0kl} + f_{0l}})\rbrack$$

We calculated the standard deviations of these cluster values by district, which would be used to elucidate the small area variation for each outcome. Finally, we generated precision-weighted estimates specific to each district for each outcome. This was calculated using Eq. (3)

3 $${\rm {exp}} \lbrack\beta _0 + \left( {v_{0kl} + f_{0l}}\right)\rbrack/ 1+{\rm {exp}} \lbrack\beta _0 + ( { v_{0kl} + f_{0l}})\rbrack$$

## Results

### Sample characteristics

Of the 2,795,894 individuals sampled in the NFHS-5, 258,808 were in the bottom 10th percentile of the wealth index, while 532,760 were in the bottom 20th percentile of the wealth index (Table 1). Of the 2,791,372 individuals living in households with complete BPL data, 1,366,554 were BPL. Finally, of the 441,293 individuals living in households with complete MDP data, 177,563 were multidimensionally poor. The percent prevalence for each outcome by state is presented in Table 1.

**Table 1:** Percent prevalence of four measures of poverty in 2021 across states and Union Territories in India.

	Bottom 10th percentile	Bottom 20th percentile	Below poverty line	Multidimensional poverty
All India	9.3	19.1	48.9	40.2
*States*				
Andhra Pradesh	1.5	3.4	91.4	22.7
Arunachal Pradesh	8.1	18.9	64.6	31.0
Assam	11.4	31.9	53.3	45.1
Bihar	18.8	38.4	57.3	67.6
Chhattisgarh	20.6	34.5	89.4	45.2
Goa	0.0	0.2	26.1	2.8
Gujarat	5.9	12.8	38.4	30.7
Haryana	0.5	1.7	24.7	22.8
Himachal Pradesh	1.1	3.9	19.9	22.5
Jharkhand	29.3	46.3	61.6	61.3
Karnataka	2.2	6.5	85.3	23.1
Kerala	0.3	0.8	44.9	2.1
Madhya Pradesh	16.1	29.9	56.9	42.8
Maharashtra	4.3	9.4	41.5	22.8
Manipur	7.5	21.5	46.5	28.1
Meghalaya	12.2	29.4	64.2	56.7
Mizoram	3.2	7.1	29.9	18.1
Nagaland	10.2	28.8	74.7	51.7
Odisha	19.4	33.5	51.6	39.6
Punjab	0.2	0.1	19.9	15.6
Rajasthan	5.6	13.1	25.5	32.4
Sikkim	0.7	2.7	46.7	17.2
Tamil Nadu	1.5	4.2	25.0	6.5
Telangana	1.5	4.8	87.8	21.5
Tripura	11.4	30.1	45.3	40.9
Uttar Pradesh	10.6	21.6	37.2	50.4
Uttarakhand	1.4	5.7	40.8	26.7
West Bengal	14.8	29.8	42.9	30.2
*Union Territories*				
Andaman & Nicobar Islands	4.5	10.0	18.9	8.3
Chandigarh	0.2	0.1	6.3	15.9
Dadra & Nagar Haveli and Daman & Diu	1.9	5.2	28.9	15.4
Jammu & Kashmir	3.4	10.1	59.3	19.6
Ladakh	1.1	9.7	62.1	14.3
Lakshadweep	0.0	0.0	34.4	2.8
Delhi	0.0	0.2	12.3	10.2
Puducherry	0.6	1.4	36.6	4.3

### Correlations between measures of wealth

**Fig. 2:**
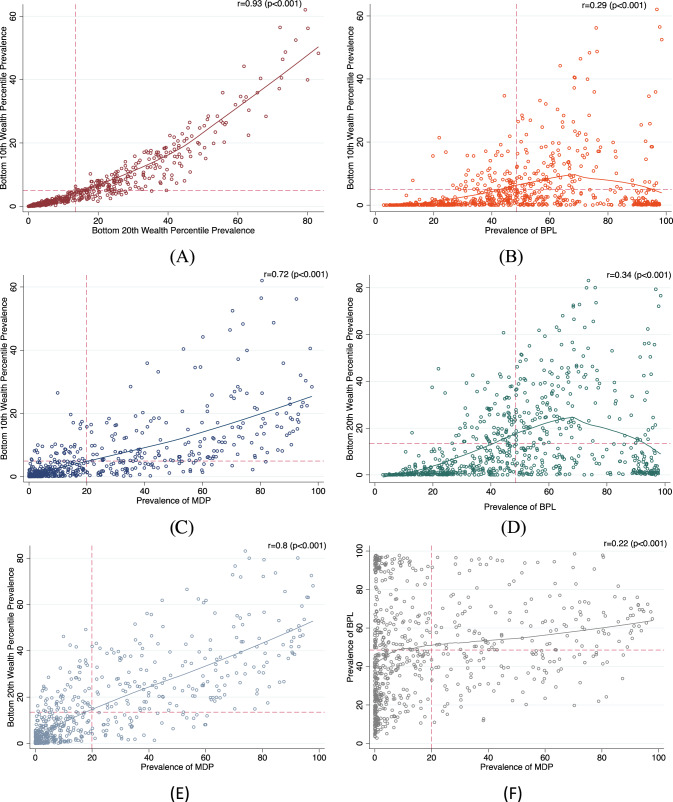
Correlation plots for the district-level prevalence between the four measures of poverty. **A** Bottom 10th wealth percentile and bottom 20th wealth percentile. **B** Bottom 10th wealth percentile and BPL. **C** Bottom 10th wealth percentile and MDP. **D** Bottom 20th wealth percentile and BPL. **E** Bottom 20th wealth percentile and MDP. **F** BPL and MDP.

We estimated the correlation values for the district means for each measure. We found strong positive correlations (0.93, *p* < 0.001; 0.72, *p* < 0.001) between the mean district percent estimates for individuals in the bottom 10th wealth percentile and individuals in the bottom 20th wealth percentile and MDP individuals. We also found a strong positive correlation (0.8, *p* < 0.001) between individuals in the bottom 20th wealth percentile and MDP individuals. We found a positive correlation (0.29, *p* < 0.001) between BPL individuals and bottom 10th wealth percentile individuals, and a positive correlation (0.34, *p* < 0.001) between BPL individuals and bottom 20th wealth percentile individuals. Finally, we found a positive correlation (0.22, *p* < 0.001) between MDP and BPL individuals. These results are presented in Fig. 2.

### Relative importance of geographic levels

**Fig. 3:**
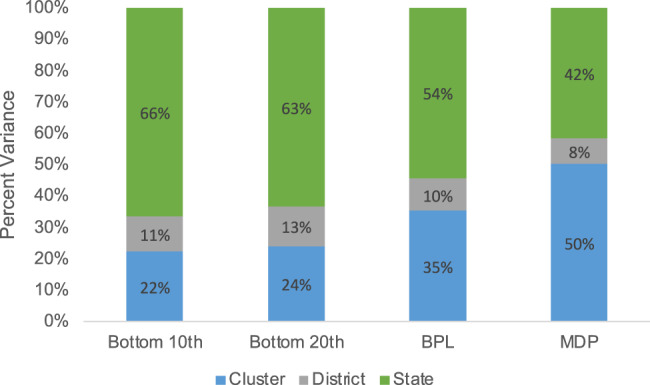
Bar graph showing the geographic variance partitioning by clusters, districts, and states for the four measures of poverty.

We found that states were the largest source of variation for individuals in the bottom 10th wealth percentile (66%), the bottom 20th wealth percentile (63%), and BPL households (54%). Clusters were the largest source of variation for MDP individuals (50%). Districts were the smallest source of variation for all four outcomes. A summary of these values is presented in Fig. 3. The variance estimates for each of the four measures of poverty are presented in Supplementary Table 1.

### Small area variation in household poverty

**Fig. 4:**
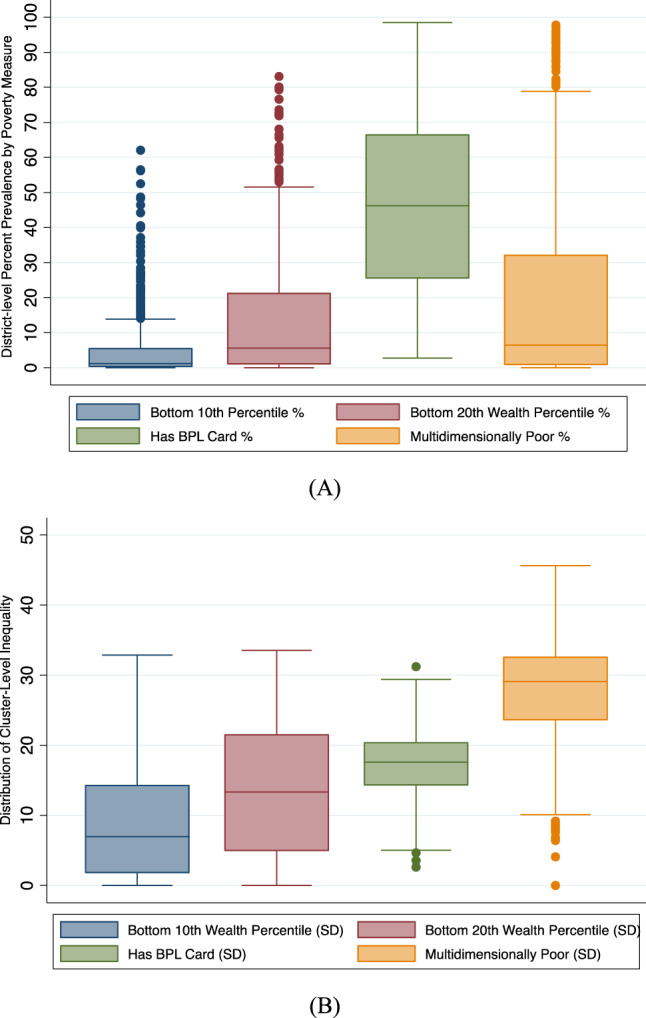
Distributions of individual prevalence by poverty measure and inequality. **A** Box plot of district-level percent of households by each poverty measure. **B** Box plot of the district-level distribution of cluster-level inequality by each poverty measure.

**Fig. 5:**
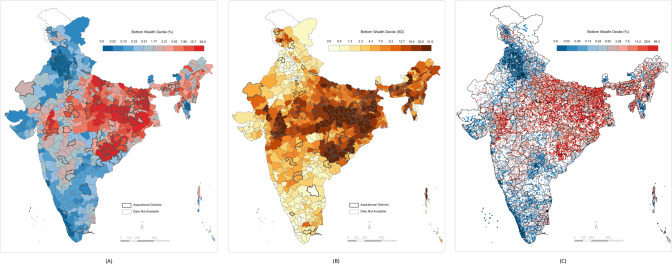
Small area maps of households in the bottom 10th wealth percentile throughout India. **A** Geographic prevalence of bottom 10th wealth percentile individuals across 640 districts in India. **B** District-level distribution of cluster-level inequality for bottom 10th wealth individuals across 640 districts in India. **C** Geographic prevalence of bottom 10th wealth percentile individuals across 30,170 clusters in India.

**Fig. 6:**
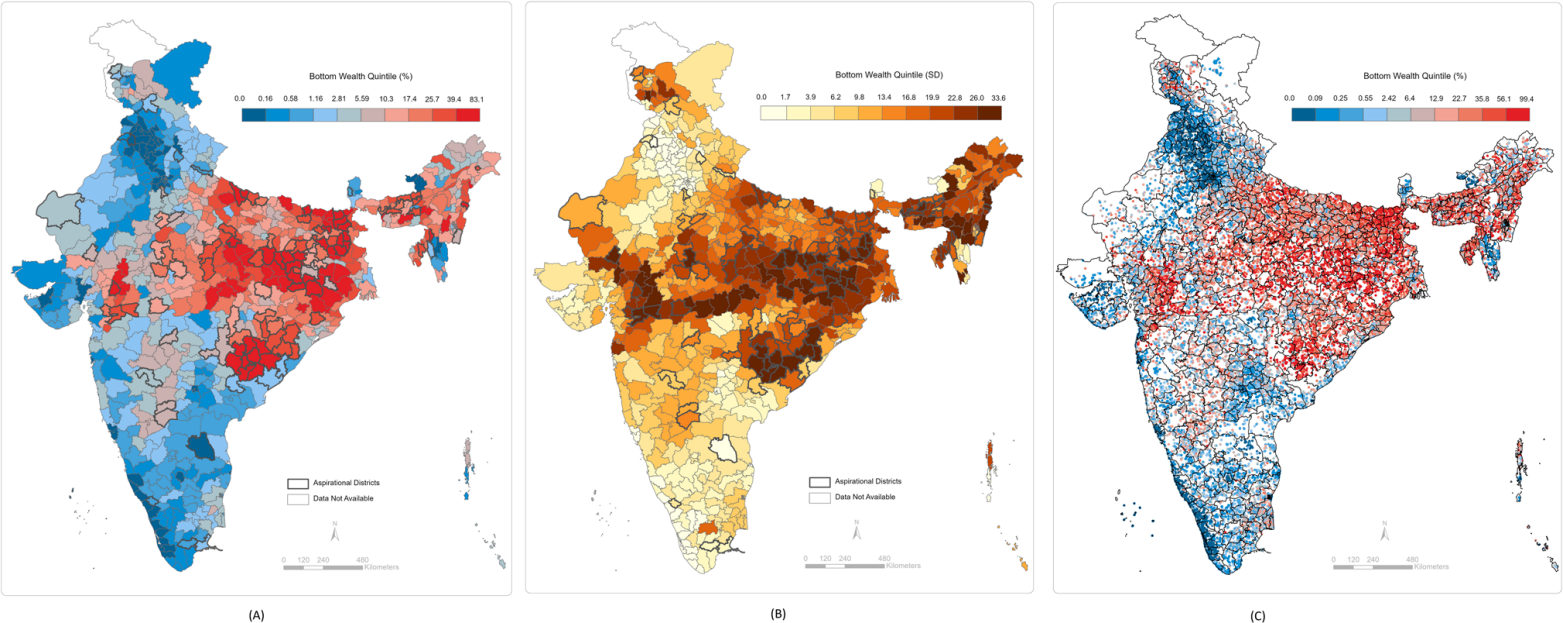
Small area maps of households in the bottom 20th wealth percentile throughout India. **A** Geographic prevalence of bottom 20th wealth percentile individuals across 640 districts in India. **B** District-level distribution of cluster-level inequality for bottom 20th wealth individuals across 640 districts in India. **C** Geographic prevalence of bottom 20th wealth percentile individuals across 30,170 clusters in India.

**Fig. 7:**
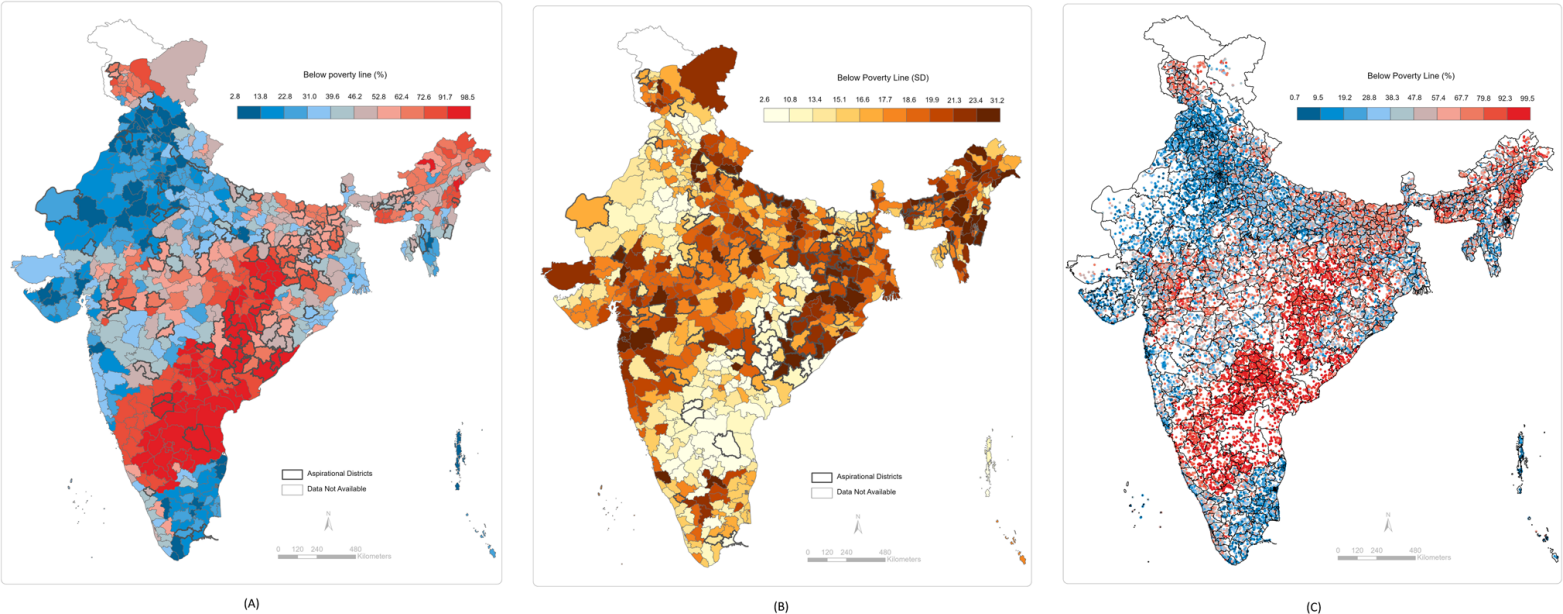
Small area maps of BPL households throughout India. **A** Geographic prevalence of BPL individuals across 640 districts in India. **B** District-level distribution of cluster-level inequality for BPL individuals across 640 districts in India. **C** Geographic prevalence of BPL individuals across 30,170 clusters in India.

**Fig. 8:**
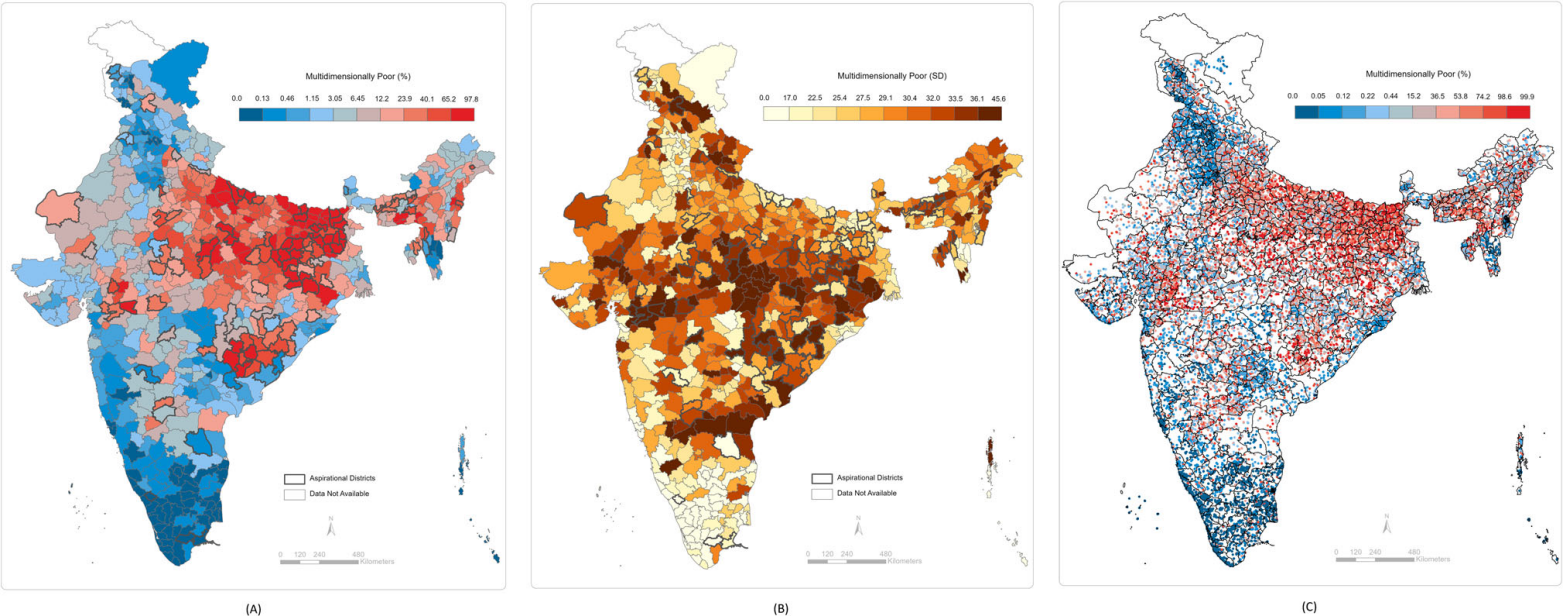
Small area maps of MDP households throughout India. **A** Geographic prevalence of MDP individuals across 640 districts in India. **B** District-level distribution of cluster-level inequality for MDP individuals across 640 districts in India. **C** Geographic prevalence of MDP individuals across 24,416 clusters in India.

**Fig. 9:**
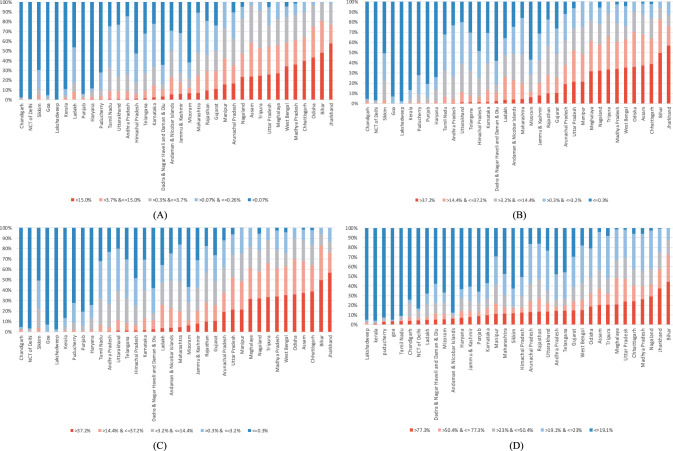
Cluster-level percent prevalence of each measure of poverty by state and Union Territory in 2021. **A** Bottom 10th wealth percentile. **B** Bottom 20th wealth percentile. **C** Below poverty line (BPL). **D** Multidimensionally poor (MDP).

We computed the standard deviations of the predicted cluster wealth index scores by each district. These values can be interpreted as the within-district and between-cluster variations in individual poverty. We computed the standard deviations of the predicted percentage of individuals in the bottom 10th and 20th wealth percentiles in each cluster by the district. The within-district between-cluster standard deviations for individuals in the bottom 10th wealth percentile ranged from 0.0004 to 32.9 with a median value of 6.9. The within-district between-cluster standard deviations for individuals in the bottom 20th wealth percentile ranged from 0.0001 to 33.6 with a median value of 14.2. The within-district between-cluster standard deviation for multidimensionally poor individuals ranged from 0.0002 to 45.6 with a median value of 29.1. Finally, within-district between-cluster standard deviation for households with BPL cards ranged from 2.6 to 31.2 with a median value of 17.6. These ranges, along with the district mean ranges, are presented in Fig. 4. The district-level predictions, between-cluster standard deviations by district, and cluster-level predictions are presented in Figs. 5–8. We also show the cluster-level prevalence of each measure of poverty by state and Union Territory in Fig. 9.

### Correlation between district percent and cluster standard deviation

**Fig. 10:**
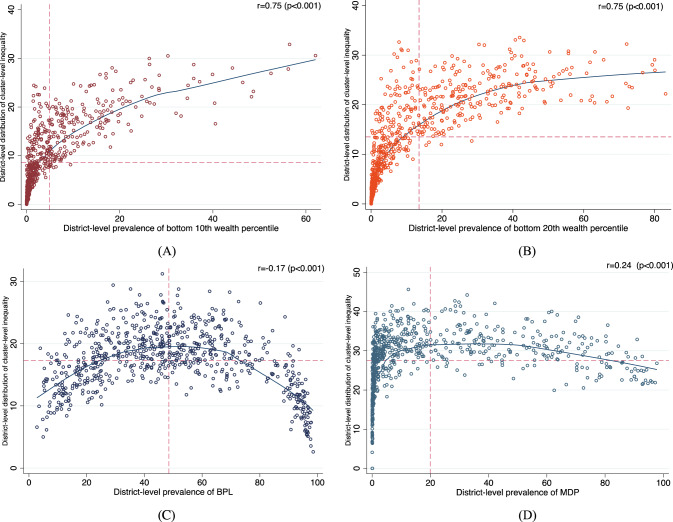
Correlation plots for the district-level prevalence and cluster-level inequality of four measures of poverty. **A** Bottom 10th wealth percentile district-level prevalence and bottom 10th wealth percentile cluster-level standard deviation. **B** Bottom 20th wealth percentile district-level prevalence and bottom 20th wealth percentile cluster-level standard deviation. **C** District-level BPL prevalence and cluster-level BPL standard deviation. **D** District-level MDP prevalence and cluster-level MDP standard deviation.

We calculated the associations between the predicted district-level percentages of individuals in the bottom 10th and 20th wealth percentiles and the cluster standard deviations. We found a significant positive correlation between the predicted district percentage of individuals in the bottom 10th percentile and the cluster standard deviation (0.75, *p* < 0.001). We also found a significant positive correlation between the predicted district percentage of individuals in the bottom 20^th^ percentile and the cluster standard deviation (0.75, *p* < 0.001). There was a significant positive correlation between the district percentage of multidimensionally poor households and the cluster standard deviation (0.24, *p* < 0.001). Finally, there was a slight negative correlation between the district percentage of households with BPL cards and the cluster standard deviation (−0.17, *p* < 0.001). These results are presented in Fig. 10.

## Discussion

This paper had four salient findings. First, we found null to moderate correlations between the district mean and SD values for all of the primary outcomes. Second, the largest share of geographic variation for each outcome was attributable either to states or clusters. Third, we found a wide range in the within-district between-cluster SD values for all four poverty measures. Furthermore, while our results show that poverty is generally clustered in north, central, and parts of east India, district-level clustering varies based on the wealth measure being analyzed. Finally, we found significant positive correlations between the percentage of individuals in the bottom 10th and 20th wealth percentiles by district and the cluster standard deviations. However, we found a significant negative correlation between the percentage of multidimensionally deprived individuals in a district and the cluster standard deviations.

There are two data limitations to this study. First, certain questions about household wealth in the NFHS are self-reported. Despite this being a possible source of measurement error, the NFHS data are widely considered to be of high quality (Corsi et al., 2012). Second, the precision-weighted estimates presented in this paper could potentially be biased by the fact that we did not adjust for any sociodemographic correlates of wealth, such as caste or household head education.

These findings could help inform anti-poverty policies in several ways. For example, our results point to the importance of considering even smaller geographic units in anti-poverty policy design. We show that a large share of the variation in poverty is attributable to clusters, highlighting the contextual influence these relatively small geographic units play on household-level outcomes. This is consistent with findings from prior studies that also show the critical role of clusters in shaping poverty outcomes in India (Kim et al., 2016). This has also been shown in the context of correlates of child undernutrition, a key indicator of household poverty (Jain et al., 2021). Thus, poverty-eradication policies such as the Aspirational Districts Program and the Mahatma Gandhi National Rural Employment Guarantee Act need to examine clusters within districts that need to be prioritized to ensure equitable advancement.

Furthermore, there is an extensive body of research documenting rising income and wealth inequality throughout India (Chancel and Piketty, 2019; Mishra and Bhardwaj, 2021; S. Subramanian and Jayaraj, 2013). Some of these studies elucidate between-district differences (Menon et al., 2018; Mohanty et al., 2016), while others have examined between-state disparities (Alkire and Seth, 2015; Anand and Thampi, 2016). Yet our findings clearly highlight the fact that variations in household wealth exist at a much smaller geographic scale. This is demonstrated by our analysis of MDP individuals and those in the bottom 10th/20th wealth percentiles, which shows that districts with a higher percentage of poor individuals tend to have greater small area variation. There are a few different explanations for widening wealth inequality between regions throughout India. Between-caste inequality, regional variations in agriculture and climate, and varying degrees of infrastructure investments are some of the possible explanations for persistent wealth inequality throughout India (Chauhan et al., 2016; Ghosh and De, 1998; Palmer-Jones and Sen, 2003, 2006; Zacharias and Vakulabharanam, 2011). Future research should explore the extent to which these factors explain the small area variations in poverty found in this study. Additionally, future research should examine how anti-poverty policies and programs can be tailored to varying within-district and between-cluster conditions so as to avoid a one size fits all approach. Doing so is important given that household wealth is associated with factors such as whether a woman has a skilled birth attendant present at delivery (Kesterton et al., 2010), children’s educational outcomes (Bacolod and Ranjan, 2008; Cashman et al., 2021), and intimate partner violence (Ackerson and Subramanian, 2008).

When viewed through the lens of social epidemiology, our results point to the difficulty in accurately measuring wealth as an indicator of socioeconomic position and its impacts on health (Braveman et al., 2001; Howe et al., 2012; Kawachi et al., 2010; Oakes and Rossi, 2003). This is emphasized by the fact that not all of the measures are clustered in the same areas throughout India, making them all different in what they might be capturing. This highlights why selecting four different primary outcomes was important given that each one measures something different. Furthermore, our results also point to the importance of measuring area indicators rather than simply individual-level measures of wealth. Previous studies have established the fact that wealth disparities and inequality are strongly associated with health (McMichael, 1999; Wilkinson and Pickett, 2006). This is important when considering multidimensional poverty given that our findings highlight that districts with a higher percentage of multidimensionally poor individuals have a greater degree of inequality. Thus, our findings pinpoint the precise districts where between-cluster inequality in poverty is most prevalent. This can help guide policy makers in terms of targeting public health and social welfare policies.

Our analysis also underscores the importance of examining the small area variations of the composite indicators of wealth given that poverty is multidimensional and is an overall deprivation in terms of assets and housing quality. Indicators such as access to safe water and sanitation and electricity are important unto themselves (Jain and Subramanian, 2018). However, unequal access to these assets can lead to deleterious health and social outcomes. Access to safe drinking water and sanitation is important for child health and psychosocial outcomes among women (Caruso et al., 2018; Fink et al., 2011; Sahoo et al., 2015). Meanwhile, household electrification is associated with increases in women’s empowerment (Samad and Zhang, 2019; Standal and Winther, 2016), which is similarly associated with improved maternal health outcomes (Grown et al., 2005; Roy and Chaudhuri, 2008). As such, the unequal distribution of these essential assets within-districts and between-clusters, could help explain small area variations in wealth-based outcomes such as child health (Chalasani, 2012; Rajpal et al., 2021). Addressing the unequal distribution of these essential assets and goods across small areas in India is particularly important in the wake of the global COVID-19 pandemic, which more than doubled the number of people in India earing $2 or less from 60 million to 134 million between 2020 and 2021 (Kochhar, 2021).

## Conclusion

In conclusion, previous research has elucidated the extent to which poverty varies between states and districts in India. There are a number of contextual factors that explain these differences. We build on this prior research to show that there also exist small area variations in poverty within districts and between clusters in India. Our results show that the degree of regional inequality in poverty depends on both the geographic level and measure of poverty being assessed. Policy makers need to be cognizant of both these factors when designing and implementing anti-poverty programs and initiatives. Doing so could help improve a number of health, social, and economic outcomes.

## Supplementary information

Supplementary File

## Data Availability

The codes used for the current study are available from the corresponding author on reasonable request.
