# Supplementary material for: Small area variations in four measures of poverty among Indian households: Econometric analysis of National Family Health Survey 2019–2021
Source: Humanit Soc Sci Commun. 2023 Jan 17;10(1):18. doi: 10.1057/s41599-023-01509-0 (PMC9843689; doi:10.1057/s41599-023-01509-0)
Supplement: Supplementary file 1 — Supplementary File [file 41599_2023_1509_MOESM1_ESM.docx]

**Supplementary table 1:** Variance estimates (95% credible intervals) for states, districts, and clusters by four measures of poverty

|  | **State** | **District** | **Cluster** |
| --- | --- | --- | --- |
| **Bottom 10th** |  |  |  |
| Variance Estimate | 11.49 (6.84, 19.21) | 1.97 (1.73, 2.26) | 3.87 (3.76, 3.98) |
| **Bottom 20th** |  |  |  |
| Variance Estimate | 8.78 (5.32, 14.16) | 1.76 (1.56, 1.97) | 3.34 (3.25, 3.42) |
| **BPL Card** |  |  |  |
| Variance Estimate | 2.21 (1.37, 3.58) | 0.42 (0.38, 0.47) | 1.44 (1.42, 1.47) |
| **MDP** |  |  |  |
| Variance Estimate | 13.35 (8.01, 21.70) | 2.54 (2.21, 2.91) | 16.17 (15.58, 16.79) |
